# Protocol-driven primary care and community linkage to reduce all-cause mortality in rural Zambia: a stepped-wedge cluster randomized trial

**DOI:** 10.3389/fpubh.2023.1214066

**Published:** 2023-08-31

**Authors:** Wilbroad Mutale, Helen Ayles, James Lewis, Samuel Bosompraph, Roma Chilengi, Margaret M. Tembo, Ab Sharp, Namwinga Chintu, Jeffrey Stringer

**Affiliations:** ^1^Department of Health Policy and Management, School of Public Health, University of Zambia, Lusaka, Zambia; ^2^Zambia AIDS Related Tuberculosis (ZAMBART), Lusaka, Zambia; ^3^MRC Tropical Epidemiology Group, London School of Hygiene and Tropical Medicine, London, United Kingdom; ^4^Centre for Infectious Disease Research in Zambia (CIDRZ), Lusaka, Zambia; ^5^Department of Biostatistics, School of Public Health, University of Ghana, Accra, Ghana; ^6^Society for Family Health in Zambia, Lusaka, Zambia; ^7^University of North Carolina, Global Women Health, Chapel Hill, NC, United States

**Keywords:** health systems, mortality, stepped wedge, quality improvement, health system strengthening

## Abstract

**Introduction:**

While tremendous progress has been made in recent years to improve the health of people living in low- and middle-income countries (LMIC), significant challenges remain. Chief among these are poor health systems, which are often ill-equipped to respond to current challenges. It remains unclear whether intensive intervention at the health system level will result in improved outcomes, as there have been few rigorously designed comparative studies. We present results of a complex health system intervention that was implemented in Zambia using a cluster randomized design.

**Methods:**

BHOMA was a complex health system intervention comprising intensive clinical training and quality improvement measures, support for commodities procurement, improved community outreach, and district level management support. The intervention was introduced as a stepped wedge cluster-randomized trial in 42 predominately rural health centers and their surrounding communities in Lusaka Province, Zambia. Baseline survey was conducted between January–May 2011, mid-line survey was conducted February–November, 2013 and Endline survey, February–November 2015.The primary outcome was all-cause mortality among those between 28 days and 60 years of age and assessed through community-based mortality surveys. Secondary outcomes included post-neonatal under-five mortality and service coverage scores. Service coverage scores were calculated across five domains (child preventative services; child treatment services; family planning; maternal health services, and adult health services). We fit Cox proportional hazards model with shared frailty at the cluster level for the primary analysis. Mortality rates were age-standardized using the WHO World Standard Population.

**Results:**

Mortality declined substantially from 3.9 per 1,000 person-years in the pre-intervention period, to 1.5 per 1,000 person-years in the post intervention period. When we compared intervention and control periods, there were 174 deaths in 49,230 person years (age-standardized rate = 4.4 per 1,000 person-years) in the control phase and 277 deaths in 74,519 person years (age-standardized rate = 4.6 per 1,000 person-years) in the intervention phase. Overall, there was no evidence for an effect of the intervention in minimally-adjusted [hazard ratio (HR) = 1.18; 95% confidence interval (CI): 0.88, 1.56; value of *p* = 0.265], or adjusted (HR = 1.12; 95% CI: 0.84, 1.49; value of *p* = 0.443) analyses.

Coverage scores that showed some evidence of changing with time since the cluster joined the intervention were: an increasing proportion of children sleeping under insecticide treated bed-net (value of *p* < 0.001); an increasing proportion of febrile children who received appropriate anti-malarial drugs (value of *p* = 0.039); and an increasing proportion of ever hypertensive adults with currently controlled hypertension (value of *p* = 0.047). No adjustments were made for multiple-testing and the overall coverage score showed no statistical evidence for a change over time (value of *p* = 0.308).

**Conclusion:**

We noted an overall reduction in post-neonatal under 60 mortality in the study communities during the period of our study, but this could not be attributed to the BHOMA intervention. Some improvements in service coverage scores were observed.

**Clinical Trial Registration:**

clinicaltrials.gov, Identifier NCT01942278.

## Introduction

There has been unprecedented progress in global health in the past 20 years mostly driven by external global health actors (1). However, progress has not been uniform, especially in lower- and middle-income countries (LMICs) where the poorest sectors of the population often face the worst outcomes (2). While globally mortality rates have decreased across all age groups over the past five decades, with the largest improvements occurring among children younger than 5 years, the current rates remain unacceptably high. In 2015, about 5.9 million children died before reaching their 5^th^ birthday; half of these resided in sub-Saharan Africa (3). Similarly, deaths among younger adults continues to increase (4). If current trends continue, more than 44 million lives will have been lost to avoidable death by 2030 (5).

The agenda to develop resilient health systems has become urgent as it has been seen as a key driver to achieving equitable and sustainable health outcomes for all. The UN Sustainable Development Goals (SDG) are ambitious and include targets to radically reduce maternal mortality, to end preventable child mortality, to end deaths from TB, AIDS and malaria, to curtail the burden of non-communicable diseases, and to achieve universal healthcare coverage (6). The services required to achieve these SDGs can only be delivered through a transformation of primary health care delivery. In many LMICs, primary health systems face inadequate financial and human resources and are overwhelmed by the needs of the ever-growing populations they serve. It is clear that system strengthening is needed, but it is unclear how best to do this approach daunting task. Zambia exemplifies the challenges facing LMIC primary health care systems (7).

The Better Health through Mentoring and Assessment (BHOMA) project was a complex public health intervention funded by the Doris Duke Charitable Foundation. BHOMA introduced a protocol-driven primary care and community linkage intervention, which aimed to improve clinical care at the primary care level and increase community demand through improved confidence in the health system. The hypothesis behind the intervention was that a systematic investment in improved clinical care would immediately improve outcomes and ultimately lead to better utilization at community level. Details of the multi-level BHOMA intervention have been published elsewhere (8). Development of the intervention was consultative and addressed most urgent local priorities, including reducing under-five and adult mortality. The primary goal was to reduce all-cause mortality in individuals between 28 days and 60 years old within the study area. We excluded neonates because the interventions necessary to improve survival in this population have been extensively studied. In this paper, we report the primary results of the BHOMA intervention focusing on post-neonatal adult mortality (PN-U60M), post neonatal under-five mortality (PN-U5M), and on service coverage.

## Methods

### The BHOMA intervention

BHOMA was conducted in 3 predominately rural districts within the Lusaka Province of Zambia. Details of the complex health systems intervention its underlying theory of change have been described in detail in prior publications (7–22). The intervention was based on the idea that the patient-provider interaction is critical to service quality and community trust, and that all aspects of an effective health system should be organized around ensuring that this encounter is effective. Toward this end, BHOMA provided support for establishment of clear clinical management protocols, improved medical record keeping, intensive clinical mentorship, on-the-job training and iterative quality improvement (QI), bolstering the commodities supply chain, and innovative linkages between clinic and community. We briefly recount its salient features here.

#### Clinical care protocols

We began by convening technical personnel within the Zambian Ministry of health to review existing guidelines for clinical care in primary settings. In general these guidelines comprised local adaptations of the WHO’s Integrated Management of Adolescent and Adult Illnesses (23), Integrated Management of Childhood Illness (24), and Emergency Obstetric and Newborn Care (25). From these guidelines we created simple, step-by-step care protocols that were supported by job aids, including checklists, wall charts, and a system of 7 clinical forms to guide clinicians through standardized patient evaluation and management. The project employed a new cadre of lay workers, known as “clinic support workers” – 2 or 3 individuals per facility – to support care delivery. These staff members assisted with patient navigation and check-in, obtained and documented vital signs, and maintained the clinic’s paper medical record system by ensuring form completion and organized filing.

#### Support for improved record keeping

The project established an organized medical records system of patient charts that were kept on-site for each patient. At the conclusion of each patient visit, these paper records were transcribed into a comprehensive, purpose-built electronic system by dedicated data entry technicians supported by the study (CommCare® https://www.dimagi.com/commcare/). The system—which used low voltage touch screen computers networked through local cellular providers—included detailed reporting functionality to monitor a set of performance indicators and clinical outcomes. These reports were available on demand to clinic leadership and comprised a key aspect of the quality improvement (QI) intervention (#3, below). At the time of registration, the electronic system assigned each patient to a specific community health worker (CHW) based upon where they live. It also kept track of open/unresolved cases and alerted CHWs through linked cell phones to patients who missed follow-up appointments so they could be traced at home (13).

#### Training and quality improvement

We formed 6 quality improvement (QI) teams, each comprising a senior clinician, pharmacist, and data technician to guide an intensive effort in refresher training and implementation of the new protocols. An initial 1-month visit to each facility included an assessment of current staffing and equipment needs, strengthening of commodities tracking and requisition procedures, strategizing for improvement in patient flow, training in the clinical protocols, and implementation of improved record keeping. After the initial implementation phase, the QI teams returned to the facility monthly for 3 visits and then quarterly to conduct structured reviews of medical records (focusing on the accuracy of diagnosis and management) and review performance metrics with the facility’s clinical officers, nurses, midwives, and other staff to develop specific goals and plans for improvement.

#### Support for commodity procurement

BHOMA provided targeted support for essential supplies and equipment needed by site-level clinicians to deliver quality care. This began with a pre-implementation assessment of each participating facility, followed by targeted equipment procurement. Part of the QI teams’ mandate was to strengthen site-level forecasting and ordering.

#### Improved community linkages

BHOMA worked to increase linkages between each facility and the community it served through active patient referral and follow-up. We recruited more than 200 CHWs who participated in 4 weeks of initial implementation training at their respective facilities. Each CHW was trained in recognition of danger signs, including when and how to refer individuals who met specific criteria to health facilities. We also trained them in how to dispense ferrous sulfate and folic acid for treatment of anemia, prescribe oral rehydration salts for child diarrhea, and give anti-malarial medication.

CHWs were each assigned to a specific zone within the catchment geography of their respective clinic. They made quarterly visits to all households in their zone where they interviewed household members and referred those in need of care. In the home, the electronic system system—installed on their mobile phones—guided the CHWs through a series of questions to which they keyed in responses. If a referral was made, the appointment was registered electronically at the corresponding clinic and not resolved in the CWH’s phone until the patient had presented. All household information, including interval patient outcomes, was transferred back to the clinic servers via mobile phone at the end of each home visit. Quarterly statistics on new pregnancies, illnesses, and deaths were aggregated and provided to neighborhood health committees to facilitate local solutions to health care access.

The CHWs also made *ad hoc* visits to patient homes when a clinic appointment had been missed. This was facilitated by the electronic health record system, which sent an alert to the mobile phone of the corresponding CWH. The system kept a running tally of each CHW’s open cases and prioritized those who were diagnosed with specified danger signs at their clinic visit.

#### District level support

Continuously throughout the project—and thus not evaluated by the randomized intervention roll-out—we provided support at the district level in governance, management, site communication, and commodities forecasting. The project supported improved implementation of existing district management tools, including the Health Management Information System (HMIS), and the District Integrated Logistics and Supplies Assessment Tool (DILSAT). We also placed a dedicated pharmacy technician at each District Health Office to strengthen the district’s ordering and supply system for equipment, supplies, diagnostics, and drugs. To ensure early ownership and integration into routine districts functions, the implementation teams worked closely with a designated district clinical lead, who was involved in most trainings and quality improvement visits.

### Intervention implementation phases

The intervention was introduced at the site level through structured on-site training and mentorship. During this implementation phase, the QI team spent 4 weeks training staff in a variety of quality-related skills and competences (Table 1). During the first 2 weeks, the QI teams conducted training on diagnosis and management of common illnesses and introduced relevant protocols for clinical case management. In week 2, we trained on patient triage and record keeping using standardized forms. In week 4, we incorporated pregnancy care. All members of staff at target health facility (clinical officers, nurses, midwives, clinic support workers, and CHWs) were engaged in the site-level training.

**Table 1:** BHOMA site-level implementation activities.

Week	Training activities	Trainees
1 and 2	Diagnosis and management of common presentations Clinical protocols	Clinical staff Clinic support workers Community health workers
3	Patient registration and triage Clinical forms Data entry Medical record keeping	Clinical staff Clinic support workers Community health workers
4	Patient registration and triage Clinical forms Data entry Medical record keeping Antenatal care, postnatal care	Clinical staff Clinic support workers Community health workers Traditional birth attendants

To assess the effectiveness of our intervention, we conducted three population-based household surveys (community surveys)—one at baseline and two at follow-up. Within each cluster households to be surveyed were randomly selected through a satellite mapping exercise where the catchment area of each facility was geographically outlined. In each cluster, squares of 900 m^2^ were marked within a 3.8 km of the health facility. Computer-generated randomization was used to determine which squares would be visited and the order of visitation. All households in randomly selected squares where the survey was started were visited until the sample size was reached.

### Trial design

**Figure 1:**
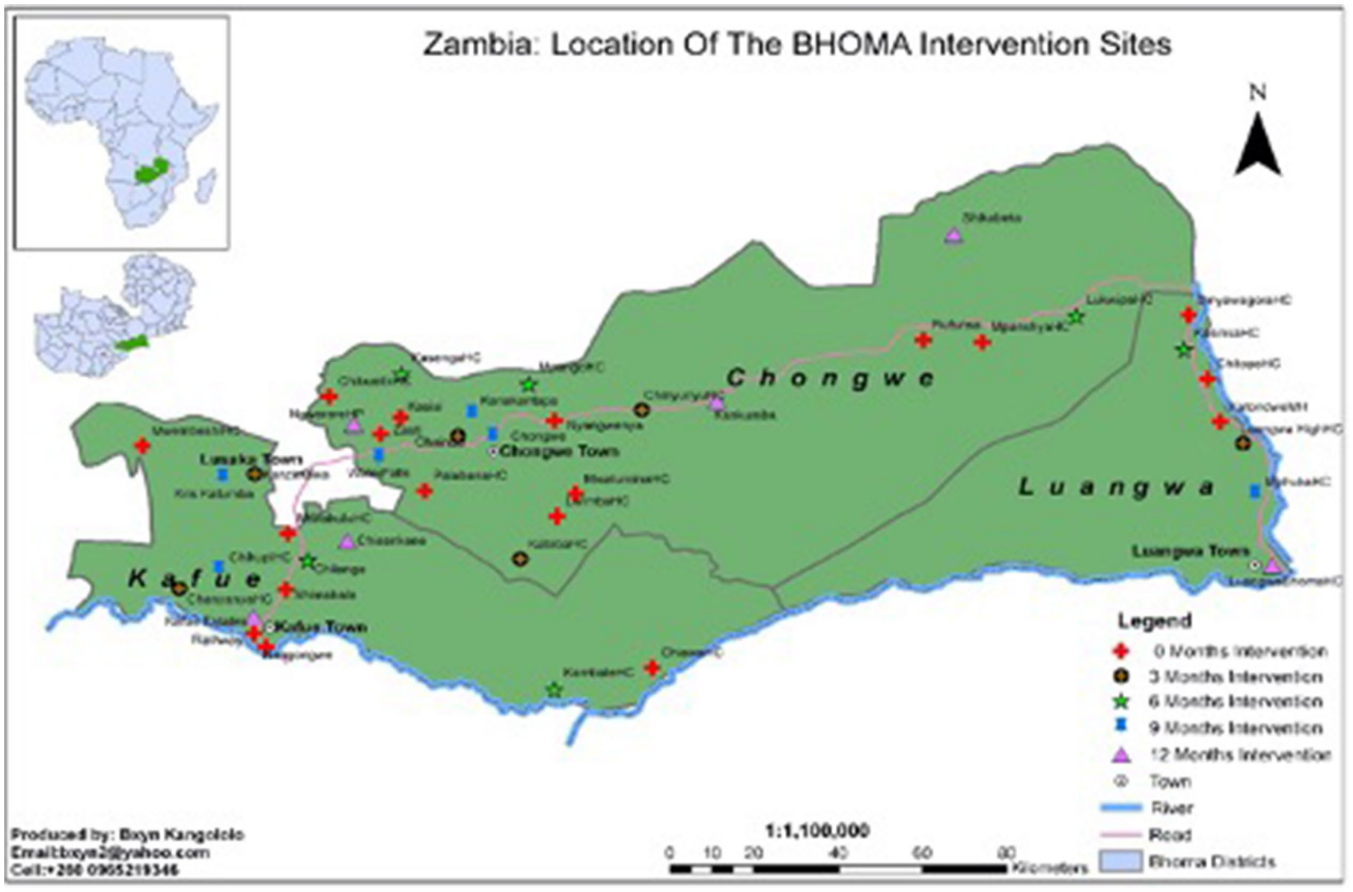
Map showing BHOMA intervention districts in Zambia.

**Figure 2:**
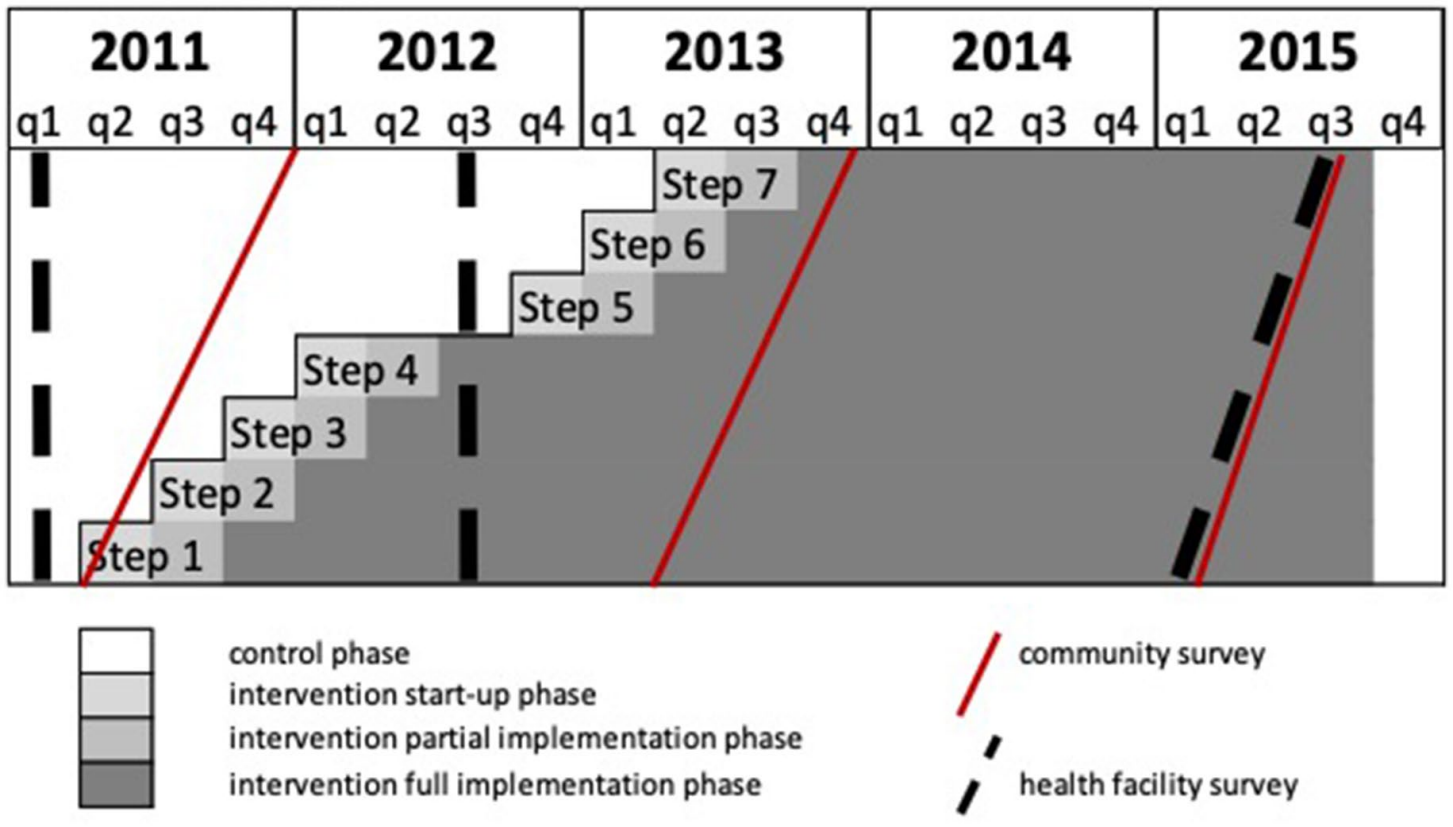
Schematic showing stepped-wedge trial design, with timing of community and health facility surveys.

The BHOMA intervention was delivered in a cluster-randomized fashion (8). The trial—formally categorized as a *stepped wedge trial of incomplete design with an implementation period*—is registered at clinicaltrials.gov (NCT01942278). Our primary unit of analysis was an implementation cluster, comprising one primary health care facility and the population that it served. All clusters were situated within three rural districts of Lusaka Province, Zambia (Figure 1). In these districts a total of 52 health facilities existed at the start of the trial and were initially eligible for inclusion. Ten clusters were excluded—three military facilities, six pilot sites, and one facility with no trained health care workers (Figure 2). The population estimates of the clusters varied from 1,501 to 44,658 people.

### Randomization and masking

Forty-two clusters were randomly assigned to start the intervention in seven steps, each step having six clusters, 3 months apart. Randomization of the clusters into the seven steps was stratified by district. At each step, three clusters from district 1, two clusters from district 2 and one cluster from district 3 were randomly assigned to start the intervention. Randomization was done by a statistician (JL) based in London and not involved in implementation, who generated a random implementation schedule using Stata version 15 (StataCorp LLC; College Station, TX). The order of roll out of the intervention could not be blinded.

### Outcomes assessment

Outcomes were measured in community and facility level surveys conducted at three time points during the study (Figure 1). Our pre-specified primary outcome—derived from community survey—was age-standardized mortality among individuals aged > 28 days and < 60 years (PN-U60M). Secondary outcomes included post-neonatal under-five mortality (PN-U5M).

The primary outcome of mortality was measured in a random sample of households (120 in the first survey and 300 in surveys 2 and 3). To estimate deaths occurring only within the cluster and to provide monthly mortality rates to allow for analysis of implementation timing in the stepped wedge roll-out, mortality rates were calculated using a retrospective household enumeration. The head of each household (or, if not available, another adult member) was asked to enumerate every person who had stayed the previous night in that household and then to go back month-by-month. We looked back 12 months for survey 1 and 24 months for surveys 2 and 3, identifying those that had been present or absent in each month. The respondent was also asked to include any individuals who had been present in the household for at least 1 month but were no longer present, either because they had left or because they had died. We collected the cause (if known) and date of all reported deaths.

The secondary outcome of PN-U5M was assessed in two ways: first by restricting the enumeration above to individuals under 5 years old and second from a separate birth history collected from women using the standard instrument employed by the Demographic and Health Survey.^1^

For each facility, Coverage scores were constructed using the methods of Victora et al. (26) We adopted 2 or 3 domains for each coverage score and calculated the coverage gap. A full list of indicators and their definitions is available in the Appendix (Supplementary Table S1). Information on the coverage indicators was collected from individual questionnaires administered to all available adults in survey 1 and in 120 randomly selected households from the 300 per cluster in surveys 2 and 3. Questionnaires were constructed to align with Demographic and Health Survey tools where possible (27).

### Sample size

The sample size was based on standard formula for parallel cluster randomized trials, adjusted for the design effect of a stepped wedge design (based on the number of steps) (28). The plan was to conduct three surveys of the 42 clusters, each recruiting 150 households per cluster, of whom 6 members were assumed aged <60 years, among whom deaths within the household in the last 12 months would be ascertained. Assuming a mortality rate for those age < 60 years of 20/1000 person years and a coefficient of variation of 0.3 there would be at least 90% power to detect a 35% reduction in mortality. Assuming a post-neonatal child mortality rate of 35/1,000 person years (corresponding to under-5 mortality of 168/1,000) and that each participating household would have an average of two children under 5 years of age, there would be at least 90% power to detect a 35% reduction in the secondary outcome of post-neonatal under-5 mortality. After completion of the baseline survey, we observed mortality rates were lower than anticipated. We thus doubled the survey sample size to 300 households per cluster in survey rounds 2 and 3.

### Statistical analysis

The primary outcome of all-cause mortality in those aged under 60 years was analyzed by constructing retrospective mortality cohorts, analyzed using a Cox proportional hazards model with shared frailty at the cluster level. The variable for intervention phase was coded as a quantitative variable with values: 0 for the control and intervention start-up phases; ½ for the intervention partial implementation phase; and 1 for the intervention full implementation phase. Sensitivity analyses were: using a Poisson model with a random effect for clustering and a categorical variable for time step; the same Poisson model, but restricted to the same time period as the Cox model; the Cox model excluding trauma deaths; Cox model excluding any persons who had not started the retrospective cohort in the community (i.e., excluding in-migrants); Cox model excluding the 3-month intervention start-up and partial implementation phases. Minimally adjusted models adjusted for district as the stratifying variable, age category (as age-adjusted rates were pre-specified) and calendar time (implicitly in the Cox model or explicitly in the Poisson model). Adjusted models also adjusted for baseline mortality rate at the cluster-level as this showed some imbalance at baseline. Mortality rates were age-standardized using the WHO World Standard Population.

Under-five mortality rates were analyzed firstly using detailed birth histories collected from all mothers in the surveys, which were used to construct retrospective cohorts. These were analyzed using Cox models as for mortality rates for those <60 years. Neonatal deaths were excluded, as were those who were alive and no longer living with the mother. Sensitivity analyses were: excluding the three-month intervention start-up and partial implementation phases; and using the primary analysis for mortality rates for those aged < 60 years, restricted to those aged under 5 years old.

Coverage scores were calculated across five domains (under-fives prevention; under-fives treatment; family planning; maternal health and adult health). Summary measures were calculated for each domain by averaging all scores within that domain; overall coverage was calculated by averaging across the five domains. As all clusters were in the control phase during the baseline survey and intervention phase at subsequent surveys, the time since the cluster joined the intervention phase was used as the exposure variable in 1 year categories. Since the order of clusters joining the intervention phase was randomized, the time since the cluster joined the intervention was also a randomized exposure variable. The coverage scores were measured at two surveys and so analysis accounted for this repeat measurement using generalized estimating equations in linear regression models.

All analyses were performed using Stata SE 15 (StataCorp, College Station, TX, United States). The trial was registered with clinicaltrials.gov, number NCT01942278.

### Ethical statement

Permission for the study was sought at community level by traditional leaders such as chiefs and “head men” or counselors in urban areas. The Ministry of Health and district health management teams gave permission for their facilities to be included in the study. For all households randomly selected to take part in the outcome assessment survey, the head of the house or another responsible adult were asked to provide written informed consent for enumeration and collection of household characteristics. Each adult (18 years and older) was asked to complete an individual level questionnaire and to give individual written informed consent. All versions of the protocol were reviewed and approved by the ethics committees of the University of Zambia, London School of Hygiene and Tropical medicine, University of Alabama at Birmingham and subsequently the University of North Carolina, Chapel Hill. The Ministry of Health also reviewed the protocol and gave permission for the study to be undertaken.

### Role of the funder

The Doris Duke Charitable Foundation (DDCF) funded the project, was involved in its planning, and monitored its progress. DDCF was not involved in implementation of the intervention or the surveys used to measure study outcomes. Neither was it involved in the interpretation of study results, the preparation of this manuscript, or the decision to submit it for publication.

## Results

Data was collected at three time points: Baseline survey was conducted between January–May 2011, mid-line survey was conducted February–November, 2013 and Endline survey, February–November 2015.

**Figure 3:**
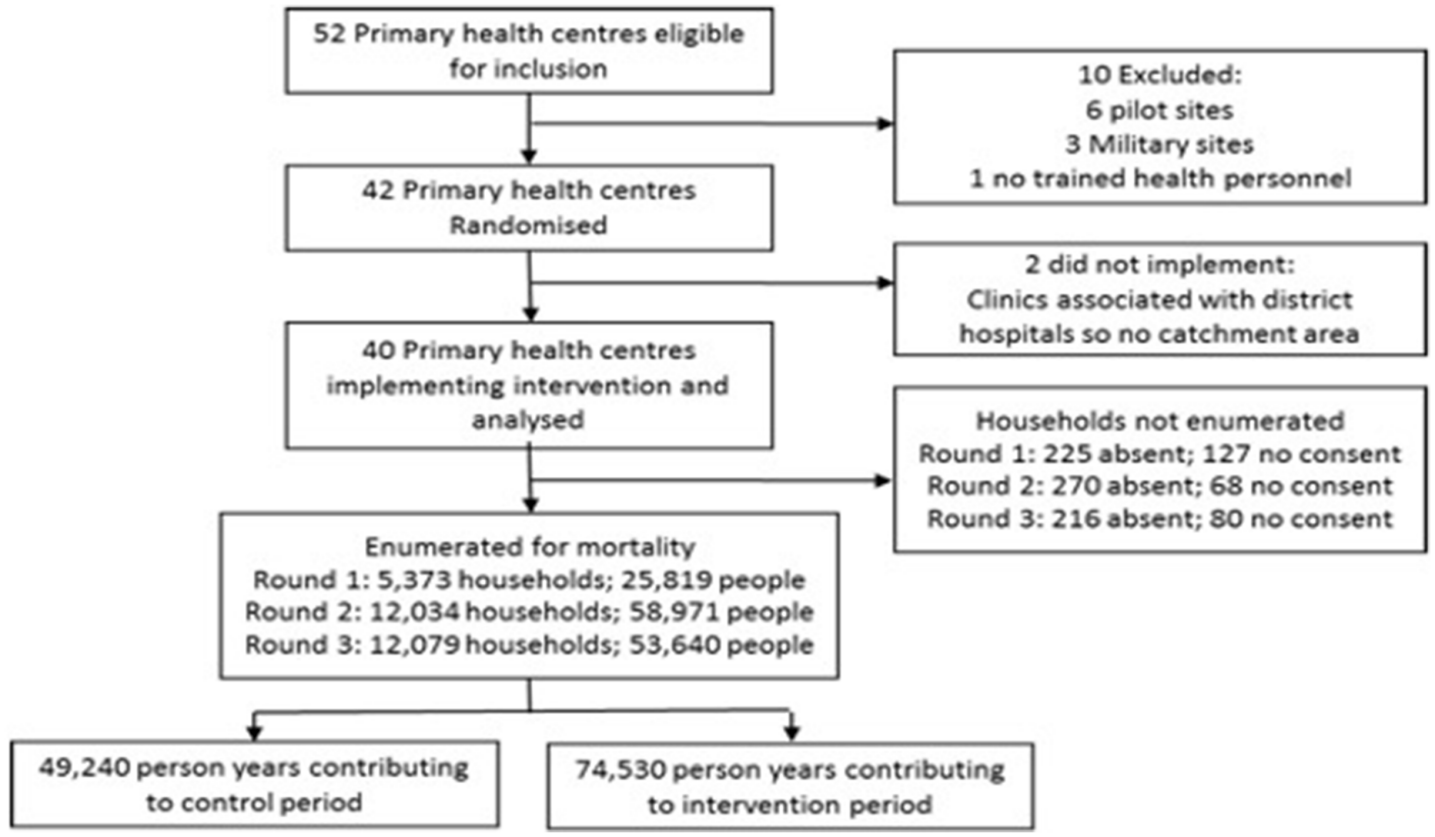
Consort diagram modified for cluster and stepped wedge.

Across the three surveys, 30,472 were randomly selected for enumeration. 29,486 households were enumerated, 711 households were absent and 275 households refused consent (Figure 3). The 29,486 households enumerated 138,430 members, giving total person years of 49,240 in the control phase and 74,530 in the intervention phase. No clusters left or joined the study during its duration. Individuals recorded in the household census in the baseline survey were 50% male and 47.5% were aged 0–14 years, 21.4% were aged 15–24 years and 31.1% were aged 25–59 years (Table 2). The population was relatively stable with 84.7% having been in the household every month in the year prior to the survey. Reasonable baseline balance was observed on key characteristics (Table 2), although the weighted average of age-standardized mortality was 3.9 per 1,000 person-years in the control phase and 5.0 per 1,000 person-years in the intervention phase. Some imbalance in the opposite direction was observed when restricted to those under 5 years old, but this estimate was based on only 19 recorded deaths.

**Table 2:** Baseline characteristics of population enumerated in the control and intervention phases of the study.

	Cluster-level means*		
	Overall	Control-phase	Intervention-phase
**Sex**			
Male	49.8%	49.8%	49.9%
Female	50.2%	50.2%	50.1%
**Age category (years)**			
0–4	17.6%	17.5%	17.6%
5–9	15.4%	16.0%	15.1%
10–14	14.5%	14.7%	14.3%
15–24	21.4%	21.2%	21.4%
25–59	31.1%	30.6%	31.6%
**Present in household for each month in prior year**			
No	15.3%	13.8%	16.4%
Yes	84.7%	86.2%	83.6%
**Mortality rate per 1,000 person years**			
Under 60 years old	4.65	3.87	4.95
Under 5 years old	4.99	5.41	4.76
**Participation in adult survey**			
No	4.4%	4.9%	4.0%
Yes	41.5%	42.1%	41.1%
Ineligible	23.5%	21.6%	24.9%
Absent at time of survey	30.1%	31.0%	29.4%
Died	0.5%	0.4%	0.5%
**Socioeconomic status of household**			
1-poorest	19.6%	18.5%	19.9%
2	19.8%	20.6%	18.8%
3	20.3%	20.8%	20.2%
4	20.2%	21.1%	19.7%
5-least poor	20.1%	19.0%	21.5%

* As all clusters were in the control phase during the baseline survey and are all in the intervention phases by surveys 2 and 3, the usual approach to presenting baseline summaries do not apply. Instead cluster-level summaries were calculated from the baseline survey and then weighted averages were calculated separately for control and intervention phases, such that the weights were the proportion of follow-up time that each cluster contributed to that phase.

**Figure 4:**
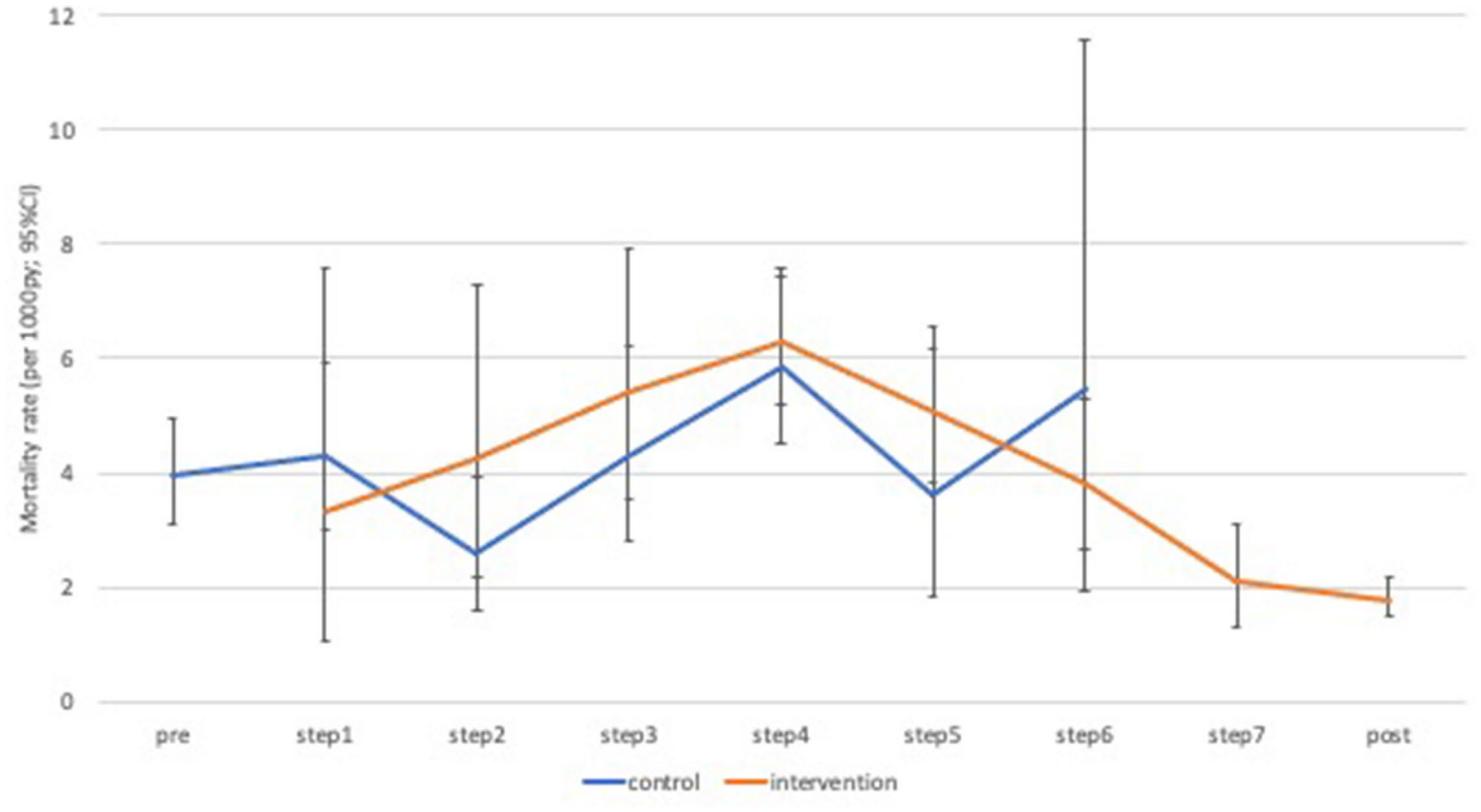
Age-standardized mortality rates by time-step and intervention phase. py, person years; CI, confidence interval.

Over the period of interest (steps one to seven), the number of deaths recorded per cluster and per intervention phase varied from zero to 17 (Supplementary Table S1). Mortality declined substantially from 3.9 per 1,000 person-years in the pre-intervention period (2011), to 1.5 per 1,000 person-years in the post intervention period (2015; Figure 4). When restricted to the formal trial period, there were 174 deaths over 49,230 person years of follow-up (age-standardized rate = 4.4 per 1,000 person-years) in the control phase compared to 277 deaths over 74,519 person years (age-standardized rate = 4.6 per 1,000 person-years) in the intervention phase (Table 3). There was no clear pattern in mortality hazard observed across the steps (Supplementary Table S2). Overall, there was no evidence for an effect of the intervention in minimally-adjusted (hazard ratio [HR] = 1.18; 95% confidence interval [CI]: 0.88, 1.56; value of *p* = 0.265), or adjusted (HR = 1.12; 95% CI: 0.84, 1.49; value of *p* = 0.443) analyses (Table 3). These results remained consistent across a range of sensitivity analyses (Table 4). There was a trend observed for an intervention effect in a planned sensitivity analysis comparing the phase of >1 year full implementation to the control phase (aHR = 0.69; 95% CI: 0.44, 1.07; value of *p* = 0.093; Table 4).

**Table 3:** Analyses of mortality rates by control versus intervention phase.

	Control phase^1^			Intervention phase^2^			Minimally adjusted analysis^3^		Adjusted analysis^4^	
Analysis	Deaths, n	Person-years of follow-up (1000)	Mortality Rate^5^ (per 1000 pyrs)	Deaths, n	Person years of follow-up (1000)	Mortality Rate^5^ (per 1000 pyrs)	aHR (95% CI)	*p*-value	aHR (95% CI)	*p*-value
Cox model	174	49,230	4.37	277	74,519	4.62	1.18 (0.88, 1.56)	0.265	1.12 (0.84, 1.49)	0.443
Poisson model	251	72,028	4.23	391	150,239	3.18	1.05 (0.80, 1.37)	0.733	0.98 (0.75, 1.27)	0.860
Poisson model, restricted to same time period as Cox	174	49,230	4.37	277	74,519	4.62	1.16 (0.87, 1.55)	0.315	1.10 (0.82, 1.48)	0.508
Cox model; non-trauma deaths only	156	49,230	3.90	243	74,519	4.08	1.15 (0.86, 1.55)	0.344	1.07 (0.80, 1.44)	0.641
Cox model; excluding in-migrants	156	47,967	4.07	228	70,772	4.10	1.11 (0.82, 1.49)	0.511	1.07 (0.79, 1.45)	0.659
Cox model; excluding 6m intervention set-up period	138	38,388	4.45	214	60,881	4.32	1.27 (0.91, 1.77)	0.152	1.21 (0.87, 1.68)	0.263

1 Control phase and intervention start-up phase combined;

2 partial and full implementation phases combined;

3 adjusted for district as the stratifying variable, age category and calendar time;

4 adjusted for district as the stratifying variable, age category, gender, calendar time and baseline mortality rate;

5 age standardised.

**Table 4:** Analysis of mortality rates by the different phases of implementation.

Phase	Deaths, n	Person-years of follow-up (1000)	Mortality Rate^1^ (per 1000 pyrs)	Minimally adjusted analysis^2^		Adjusted analysis^3^	
				aHR (95% CI)	*p*-value	aHR (95% CI)	*p*-value
Control	138	38,388	4.45	1	0.091	1	0.047
Intervention start-up and partial implementation	99	24,564	5.12	1.16 (0.87, 1.55)	0.306	1.13 (0.85, 1.51)	0.410
Full implementation <1 y	182	53,152	4.19	1.05 (0.77, 1.42)	0.771	0.98 (0.73, 1.33)	0.921
Full implementation ≥1 y	102	55,873	2.27	0.77 (0.49, 1.19)	0.236	0.69 (0.44, 1.07)	0.093

1 age standardised;

2 adjusted for district as the stratifying variable, age category and calendar time;

3 adjusted for district as the stratifying variable, age category, gender, calendar time and baseline mortality rate.

Whether calculated from the birth cohorts or by restricting the primary analysis to those under-5 years, there was no evidence for an impact on mortality among under-fives (Table 4). However, in one sensitivity analysis there was weak evidence for a negative impact of the intervention on mortality among under-fives (aHR = 2.21; 95% CI: 1.01, 4.84; value of *p* = 0.048; Table 5).

**Table 5:** Analyses of mortality rates among those aged under five years by control versus intervention phase.

	Control phase^1^			Intervention phase^2^			Minimally adjusted analysis^3^		Adjusted analysis^4^	
Analysis	# deaths	Person years	Mortality Rate (per 1000 pyrs)	# deaths	Person years	Mortality Rate (per 1000 pyrs)	aHR (95% CI)	*p*-value	aHR (95% CI)	*p*-value
Birth history	33	5,373	6.14	30	5,647	5.31	1.52 (0.77, 3.00)	0.232	1.44 (0.73, 2.86)	0.295
Birth history; excluding 6m intervention set-up period	25	4,347	5.75	26	4,420	5.88	2.43 (1.12, 5.28)	0.025	2.21 (1.01, 4.84)	0.048
Household census, restricted to <5years	33	6,374	5.18	59	9,757	6.05	1.34 (0.73, 2.46)	0.344	1.09 (0.60, 1.98)	0.770

1 Control phase and intervention start-up phase combined;

2 partial and full implementation phases combined;

3 adjusted for district as the stratifying variable, age category (in years) and calendar time;

4 adjusted for district as the stratifying variable, age category (in years), gender, calendar time and baseline mortality rate among those <60 years.

**Table 6:** Analysis of coverage by the duration of full implementation.

Period of full implementation phase	Number of clusters	Coverage		Unadjusted analysis ^1^^,^^3^		Adjusted analysis^2^^,^^3^	
		mean	standard deviation	difference (95% CI)	*p*-value	difference (95% CI)	*p*-value
Control	40	45.6%	6.0%				
0-11 months	22	44.5%	6.4%	0	0.321	0	0.308
12-23 months	24	45.9%	7.1%	1.8% (-1.8%, 5.4%)		1.4% (-2.0%, 4.8%)	
24-35 months	18	47.4%	5.4%	2.6% (-0.2%, 5.3%)		2.7% (-0.1%, 5.4%)	
36+ months	16	46.2%	6.9%	2.2% (-1.7%, 6.1%)		1.7% (-1.9%, 5.4%)	

1 Adjusted for district as the stratifying variable;

2 adjusted for district as the stratifying variable and baseline coverage;

3 linear regression model with generalised estimating equations to give robust standard errors accounting for two measurements of each cluster.

Some, but not all, coverage scores showed statistical evidence of improvement as a result of the intervention, including: an increasing proportion of children sleeping under insecticide treated bed-net (value of *p* < 0.001); an increasing proportion of febrile children who received appropriate anti-malarial drugs (value of *p* = 0.039); and an increasing proportion of ever hypertensive adults with currently controlled hypertension (value of *p* = 0.047; Supplementary Tables S3 and S4). No adjustments were made for multiple-testing and the overall coverage score showed no statistical evidence for a change over time (value of *p* = 0.308) (Table 6).

## Discussion

Our study findings indicate mixed results for the BHOMA health system intervention. While we observed a general decline in post-neonatal under 60 mortality (from 3.9 to 1.5 per 1,000 person-years) over the period of surveillance, we were unable to directly attribute this reduction to our intervention. We similarly did not observe an effect on post-neonatal under 5 mortality. We did, however, observe significant improvement in facility-level coverage scores for a number of key indicators.

Our findings are consistent with prior work in Rwanda and Ghana (29, 30), where health system interventions have been associated with substantial improvement in process indicators. Most prior studies have employed “before and after” comparisons, which require caution in their interpretation because potential bias may be introduced through secular trends. We attempted to mitigate this bias through a cluster randomized stepped wedge design, but cannot completely rule out such an effect.

Another issue to consider when interpreting these findings is the complex and dynamic nature of the intervention that was implemented (11, 12). Our intervention attempted simultaneous intervention upon multiple “building blocks” (7, 11) of the health system, some of which occurred at a level above that of the randomization. For instance, the efforts we made to strengthen management capacity at the district level could not be evaluated through the stepped wedge randomization, and may have biased our outcomes toward the null. Furthermore, the complexity and scope of our multi-faceted intervention may have invited opportunistic adaptation at the district or provincial level with some “leakage” of benefits to study sites prior to formal intervention. For example, our work at the district level led to shared benefit across the control sites thus potentially diluting expected or desired effect (14). We also supported the district planning and supply chains, which could have benefited control sites, even before we introduced the intervention (14, 30). Trials are embedded within an already existing health system which tend to be hierarchical, with primary centers being supervised by districts which are in turn are supervised by provincial health teams who report to the national level. This is true for the Zambian health system. This hierarchical interdependence could potentially have introduced an element of complexity which statistical models in this analysis may not account for (31).

We used retrospective assessment of child survival *via* birth history analysis, which has potential bias relating to event omission or event displacement recall biases. In addition, there are well-described cultural beliefs in Zambia that prevent people from talking about stillbirth and child death (32). This phenomenon could have contributed to under reporting particularly for infants (31).

An important limitation for our study is inherent in its stepped-wedged design (33). As in our study, stepped-wedge cluster randomized trials are often conducted on a large scale and are therefore sensitive to external challenges that cannot directly be influenced by the researchers such as changes in the local context (33). Our study communities experienced several competing interventions during the study period (7, 11, 14). These could have impacted on the outcome measures we intended (7) (33)

Our intervention may also have been dependent on the temporal nature of the intervention; it may have taken time for the improvements to fully manifest. Delay in achieving the desired effect could result in loss of power for the stepped wedge design, especially if the intervention is not fully effective in the time interval in which the evaluation was conducted 34. Such delays can be caused by a slower than expected intervention rollout or by intrinsic lag between introduction of the intervention and its effect on the outcome. Our trial had two challenges that fit well with this observation. Firstly, we had a 6-month delay in introducing the 5th of the 7 clusters due to administrative issues with funding. A second challenge was related to the outcome we aimed to improve. Improvements in all-cause mortality may have taken more than the 3 months we used per implementation step to manifest. Perhaps longer implementation steps and longer follow-up time would have yielded different results 34. The observation that a few short-term outcomes in our study, such as coverage scores, showed significant improvement across the steps, may support this argument. We have similarly reported positive health systems impact of the BHOMA intervention in the corresponding publication.

Our findings have public health and research significance, first we utilized a gold standard randomized approach to evaluate this multi-faceted intervention. Despite the challenges outlined, this is one of the few complex health system interventions that have employed both a randomized design and mortality outcomes to evaluate efficacy. The fact that our chosen outcome measure of mortality was not demonstrated, but instead demonstrated a change in the process indicators such as coverage and health system indicators (Mutale, BHOMA paper 2), indicate that allowing longer term follow-up is crucial to making definitive conclusion. It will be important for future studies to consider varying periods of follow-up time to in order to estimate minimum time required to establish effect of similar large health system interventions on mortality. Our study had a very short follow-up time and is therefore not able to estimate such lead time. Other modern approaches such as modeling the period and effect size should be considered in future studies.

Finally, the study clearly demonstrate the importance of carefully choosing indicators and allowing sufficient follow-up time when evaluating complex health system interventions. Our findings may be generalizable to other settings but we caution that follow-up time need to be sufficient and that chosen indicators must be realistic within the study time frame in order to demonstrate meaningful health system impact.

## Data availability statement

The original contributions presented in the study are included in the article/supplementary material, further inquiries can be directed to the corresponding author.

## Ethics statement

The studies involving human participants were reviewed and approved by University of Zambia Bioethics Committee. The patients/participants provided their written informed consent to participate in this study.

## Author contributions

WM, HA, JL, SB, RC, MT, AS, NC, and JS participated in the design and implementation of the intervention. JL and SB conducted the analysis. WM drafted the manuscript. All authors contributed to the article and approved the submitted version.

## Funding

This work was supported by Doris Duke Charitable Foundation (DDCF).

## Conflict of interest

The authors declare that the research was conducted in the absence of any commercial or financial relationships that could be construed as a potential conflict of interest.

## Publisher’s note

All claims expressed in this article are solely those of the authors and do not necessarily represent those of their affiliated organizations, or those of the publisher, the editors and the reviewers. Any product that may be evaluated in this article, or claim that may be made by its manufacturer, is not guaranteed or endorsed by the publisher.

## Footnotes

^1^https://dhsprogram.com
