# Supplementary material for: Protocol-driven primary care and community linkage to reduce all-cause mortality in rural Zambia: a stepped-wedge cluster randomized trial
Source: Front Public Health. 2023 Aug 31;11:1214066. doi: 10.3389/fpubh.2023.1214066 (PMC10505962; doi:10.3389/fpubh.2023.1214066)
Supplement: Supplementary file 1 [file Table_1.docx]

Table S1: Cluster specific mortality rates by control versus intervention phase.

|  | Control phase ^1^ | | | Intervention phase ^2^ | | |
| --- | --- | --- | --- | --- | --- | --- |
|  | Deaths, n | Person-years of follow-up (1000) | Mortality Rate ^3^ (per 1000 pyrs) | Deaths, n | Person years of follow-up (1000) | Mortality Rate ^3^ (per 1000 pyrs) |
| District A |  |  |  |  |  |  |
| Cluster 1 |  |  |  | 4 | 2.16 | 1.68 |
| Cluster 2 |  |  |  | 13 | 2.64 | 5.10 |
| Cluster 3 |  |  |  | 10 | 3.56 | 3.33 |
| Cluster 4 | 0 | 0.24 | 0.00 | 7 | 2.48 | 3.42 |
| Cluster 5 | 0 | 0.27 | 0.00 | 12 | 2.72 | 5.16 |
| Cluster 6 | 1 | 0.31 | 3.10 | 7 | 2.85 | 2.92 |
| Cluster 7 | 3 | 0.54 | 6.85 | 14 | 1.79 | 11.22 |
| Cluster 8 | 3 | 0.68 | 3.67 | 11 | 2.43 | 5.02 |
| Cluster 9 | 1 | 0.78 | 0.75 | 7 | 2.69 | 3.25 |
| Cluster 10 | 5 | 0.90 | 6.31 | 17 | 1.76 | 12.57 |
| Cluster 11 | 4 | 1.24 | 3.07 | 3 | 2.41 | 1.61 |
| Cluster 12 | 5 | 1.36 | 4.42 | 10 | 2.61 | 4.87 |
| Cluster 13 | 10 | 2.13 | 6.49 | 16 | 1.29 | 17.50 |
| Cluster 14 | 11 | 2.24 | 7.01 | 10 | 1.33 | 9.55 |
| Cluster 15 | 5 | 2.26 | 2.83 | 3 | 0.83 | 3.78 |
| Cluster 16 | 4 | 2.53 | 1.91 | 4 | 0.85 | 6.10 |
| Cluster 17 | 6 | 2.68 | 2.54 | 2 | 0.95 | 3.14 |
| Cluster 18 | 5 | 2.30 | 2.87 | 0 | 0.43 | 0.00 |
| Cluster 19 | 6 | 2.65 | 3.46 | 0 | 0.44 | 0.00 |
| Cluster 20 | 7 | 2.74 | 3.14 | 5 | 0.53 | 11.96 |
| District B |  |  |  |  |  |  |
| Cluster 1 |  |  |  | 13 | 2.89 | 5.50 |
| Cluster 2 |  |  |  | 9 | 3.62 | 2.93 |
| Cluster 3 | 2 | 0.31 | 7.74 | 7 | 2.68 | 3.66 |
| Cluster 4 | 0 | 0.32 | 0.00 | 14 | 2.82 | 5.88 |
| Cluster 5 | 2 | 0.64 | 1.99 | 5 | 2.18 | 2.76 |
| Cluster 6 | 0 | 0.74 | 0.00 | 9 | 2.41 | 4.43 |
| Cluster 7 | 3 | 1.04 | 2.76 | 6 | 1.92 | 3.57 |
| Cluster 8 | 4 | 1.13 | 4.48 | 10 | 2.04 | 5.96 |
| Cluster 9 | 11 | 1.72 | 9.20 | 1 | 1.04 | 1.42 |
| Cluster 10 | 7 | 2.11 | 4.38 | 1 | 1.14 | 1.15 |
| Cluster 11 | 11 | 2.19 | 5.61 | 7 | 0.77 | 11.60 |
| Cluster 12 | 6 | 2.60 | 3.00 | 2 | 0.85 | 3.00 |
| Cluster 13 | 10 | 2.16 | 5.26 | 0 | 0.33 | 0.00 |
| Cluster 14 | 12 | 2.18 | 6.62 | 0 | 0.37 | 0.00 |
| District C |  |  |  |  |  |  |
| Cluster 1 |  |  |  | 14 | 3.27 | 5.04 |
| Cluster 2 | 3 | 0.36 | 12.62 | 6 | 3.08 | 2.51 |
| Cluster 3 | 3 | 0.67 | 6.26 | 4 | 2.38 | 2.43 |
| Cluster 4 | 4 | 0.98 | 3.82 | 8 | 1.85 | 6.38 |
| Cluster 5 | 10 | 1.90 | 7.17 | 3 | 1.24 | 3.33 |
| Cluster 6 | 10 | 2.36 | 5.91 | 3 | 0.89 | 3.46 |

^1^ Control phase and intervention start-up phase combined; ^2^ partial and full implementation phases combined; ^3^ age standardised.
