# Supplementary material for: Protocol-driven primary care and community linkage to reduce all-cause mortality in rural Zambia: a stepped-wedge cluster randomized trial
Source: Front Public Health. 2023 Aug 31;11:1214066. doi: 10.3389/fpubh.2023.1214066 (PMC10505962; doi:10.3389/fpubh.2023.1214066)
Supplement: Supplementary file 2 [file Table_2.docx]

Table S2: Time-step specific mortality rates by control versus intervention phase.

|  | Control phase ^1^ | | | Intervention phase ^2^ | | | Minimally adjusted analysis ^3^ | |
| --- | --- | --- | --- | --- | --- | --- | --- | --- |
| Time step | Deaths, n | Person-years of follow-up (1000) | Mortality Rate ^3^ (per 1000 pyrs) | Deaths, n | Person years of follow-up (1000) | Mortality Rate ^3^ (per 1000 pyrs) | HR (95% CI) | p-value |
|  |  |  |  |  |  |  |  |  |
| Pre | 77 | 22.80 | 3.94 |  |  |  |  |  |
| 1 | 38 | 10.27 | 4.30 | 5 | 1.79 | 2.79 | 0.55 (0.10, 2.96) ^4^ | 0.490 |
| 2 | 22 | 10.06 | 2.59 | 12 | 3.61 | 3.32 | 1.99 (0.88, 4.51) | 0.099 |
| 3 | 27 | 7.85 | 4.28 | 27 | 5.95 | 4.54 | 1.25 (0.73, 2.16) ^4^ | 0.415 |
| 4 | 69 | 15.07 | 5.85 | 115 | 22.39 | 5.14 | 1.01 (0.73, 1.40) | 0.948 |
| 5 | 12 | 4.54 | 3.60 | 59 | 14.38 | 4.10 | 1.56 (0.72, 3.42) | 0.262 |
| 6 | 6 | 1.45 | 5.44 | 35 | 12.14 | 2.88 | 0.42 (0.17, 1.06) | 0.067 |
| 7 |  |  |  | 24 | 14.26 | 1.68 | 0.51 (0.04, 7.35) | 0.620 |
| Post |  |  |  | 114 | 75.72 | 1.51 |  |  |

^1^ Control phase and intervention start-up phase combined; ^2^ partial and full implementation phases combined; ^3^ adjusted for district as the stratifying variable, age category and calendar time with shared frailty at the cluster level; ^4^ robust standard errors adjust for clustering as shared frailty model did not converge.
