# Supplementary material for: Protocol-driven primary care and community linkage to reduce all-cause mortality in rural Zambia: a stepped-wedge cluster randomized trial
Source: Front Public Health. 2023 Aug 31;11:1214066. doi: 10.3389/fpubh.2023.1214066 (PMC10505962; doi:10.3389/fpubh.2023.1214066)
Supplement: Supplementary file 3 [file Table_3.docx]

Table S3: Coverage scores separately and by domain, across the three surveys.

|  | Baseline survey | | | Survey 2 | | | Survey 3 | | |
| --- | --- | --- | --- | --- | --- | --- | --- | --- | --- |
|  | n/N | Cluster-level mean | 95% CI ^1^ | n/N | Cluster-level mean | 95% CI ^1^ | n/N | Cluster-level mean | 95% CI ^1^ |
| **Under-fives prevention** |  |  |  |  |  |  |  |  |  |
| Children aged 12-23 months who are fully immunized against DTP3 | 478/711 | 64.3% | (58.9%, 69.8%) | 243/471 | 51.9% | (46.4%, 57.4%) | 256/  467 | 51.9% | (45.0%, 58.8%) |
| Children 12-23 months vaccinated with BCG | 492/711 | 66.2% | (61.0%, 71.4%) | 253/471 | 53.4% | (47.6%, 59.2%) | 267/  467 | 54.3% | (47.0%, 61.5%) |
| Children aged 12-23 months who are fully immunized against measles | 468/711 | 63.0% | (57.5%, 68.5%) | 228/471 | 48.3% | (42.7%, 54.0%) | 236/  467 | 48.0% | (41.7%, 54.2%) |
| Children sleeping under insecticide treated bed-net | 1505/3666 | 41.2% | (37.0%, 45.4%) | 3283/8804 | 38.5% | (32.0%, 44.9%) | 4144/7520 | 56.0% | (49.1%, 62.8%) |
| Under-fives prevention score |  | 59.8% | (55.5%, 64.2%) |  | 48.0% | (43.7%, 52.4%) |  | 52.5% | (47.9%, 57.1%) |
| **Under-fives treatment** |  |  |  |  |  |  |  |  |  |
| Children receiving correct management for acute diarrhoea | 257/434 | 58.6% | (52.3%, 64.9%) | 161/260 | 63.5% | (54.5%, 72.4%) | 133/216 | 61.0% | (52.0%, 70.0%) |
| Children with suspected pneumonia receiving antibiotics | 75/109 | 68.4% | (55.0%, 81.8%) | 53/75 | 64.5% | (49.6%, 79.4%) | 28/33 | 84.2% | (66.2%, 102.2%) |
| Febrile children who received appropriate anti-malarial drugs | 110/339 | 29.8% | (22.0%, 37.6%) | 85/246 | 29.9% | (19.5%, 40.3%) | 34/141 | 21.0% | (11.2%, 30.9%) |
| Under-fives treatment score |  | 50.0% | (44.7%, 55.2%) |  | 51.8% | (45.5%, 58.0%) |  | 50.4% | (42.4%, 58.3%) |
| **Family planning** |  |  |  |  |  |  |  |  |  |
| Need for family planning satisfied | 968/1771 | 54.5% | (51.3%, 57.7%) | 905/1526 | 58.7% | (56.1%, 61.3%) | 858/1335 | 63.9% | (60.1%, 67.7%) |
| Information on family planning provided | 3117/7193 | 43.5% | (39.9%, 47.2%) | 2749/6221 | 44.2% | (41.1%, 47.2%) | 2539/5820 | 43.3% | (39.5%, 47.0%) |
| Family planning score |  | 49.0% | (46.4%, 51.6%) |  | 51.4% | (49.1%, 53.7%) |  | 53.6% | (50.9%, 56.3%) |
| **Maternal health** |  |  |  |  |  |  |  |  |  |
| Antenatal clinic attendance at least four times | 233/463 | 51.8% | (45.5%, 58.2%) | 184/357 | 56.9% | (48.7%, 65.1%) | 230/389 | 58.1% | (50.1%, 66.0%) |
| Births attended by skilled caregiver | 359/529 | 68.2% | (62.0%, 74.3%) | 335/424 | 79.1% | (73.9%, 84.3%) | 375/459 | 83.0% | (77.9%, 88.0%) |
| Women attending postnatal care | 101/359 | 25.1% | (18.4%, 31.9%) | 81/327 | 20.3% | (13.3%, 27.4%) | 129/374 | 24.9% | (17.3%, 32.4%) |
| Maternal health score |  | 48.4% | (44.2%, 52.6%) |  | 52.0% | (48.7%, 55.3%) |  | 55.0% | (50.8%, 59.2%) |
| **Adult health** |  |  |  |  |  |  |  |  |  |
| Adults tested for HIV in last year | 2360/6811 | 34.6% | (32.1%, 37.1%) | 2318/5799 | 40.5% | (37.7%, 43.2%) | 2087/5391 | 39.0% | (36.5%, 41.5%) |
| TB suspects correctly screened for TB | 20/171 | 14.5% | (7.1%, 21.9%) | 9/85 | 10.4% | (2.8%, 18.0%) | 5/60 | 13.3% | (0.4%, 26.3%) |
| Ever hypertensive adults with currently controlled hypertension | 181/1594 | 11.8% | (9.1%, 14.5%) | 135/1266 | 10.7% | (8.3%, 13.1%) | 166/1167 | 14.7% | (11.9%, 17.6%) |
| Adult health score |  | 20.7% | (17.8%, 23.6%) |  | 21.1% | (18.3%, 23.8%) |  | 23.9% | (20.7%, 27.2%) |
| **Overall coverage score** |  | 45.6% | (43.7%, 47.5%) |  | 44.9% | (42.9%, 46.8%) |  | 47.0% | (44.9%, 49.2%) |

^1^ 95% CI = 95% confidence interval of the cluster-level mean.
