# Supplementary material for: Protocol-driven primary care and community linkage to reduce all-cause mortality in rural Zambia: a stepped-wedge cluster randomized trial
Source: Front Public Health. 2023 Aug 31;11:1214066. doi: 10.3389/fpubh.2023.1214066 (PMC10505962; doi:10.3389/fpubh.2023.1214066)
Supplement: Supplementary file 4 [file Table_4.docx]

Table S4: Change in coverage scores by time since cluster joined the intervention.

|  | Year 2 versus year 1 | | Year 3 versus year 1 | | Year 4 versus year 1 | | Overall | |
| --- | --- | --- | --- | --- | --- | --- | --- | --- |
|  | Difference in coverage  (95% CI) | p-value ^1^ | Difference in coverage  (95% CI) | p-value ^1^ | Difference in coverage  (95% CI) | p-value ^1^ | p-value ^1^ |  |
| **Under-fives prevention** |  |  |  |  |  |  |  |  |
| Children aged 12-23 months who are fully immunized against DTP3 | -0.6 (-11.1, 9.9) | 0.908 | 1.0 (-11.3, 13.3) | 0.873 | -2.5 (-14.9, 9.8) | 0.688 | 0.790 |  |
| Children 12-23 months vaccinated with BCG | 4.1 (-6.9, 15.1) | 0.467 | 1.7 (-11.4, 14.9) | 0.799 | -2.1 (-13.8, 9.6) | 0.724 | 0.692 |  |
| Children aged 12-23 months who are fully immunized against measles | 1.8 (-9.0, 12.7) | 0.741 | 2.1 (-10.8, 14.9) | 0.750 | -1.7 (-14.3, 10.9) | 0.791 | 0.847 |  |
| Children sleeping under insecticide treated bed-net | 13.0 (8.4, 17.7) | <0.001 | 26.1 (19.8, 32.3) | <0.001 | 34.4 (26.9, 41.8) | <0.001 | <0.001 |  |
| Under-fives prevention score | 6.4 (-1.7, 14.5) | 0.119 | 8.3 (-0.8, 17.5) | 0.074 | 7.6 (-1.6, 16.7) | 0.104 | 0.065 |  |
| **Under-fives treatment** |  |  |  |  |  |  |  |  |
| Children receiving correct management for acute diarrhoea | 2.4 (-14.8, 19.6) | 0.787 | -4.2 (-17.6, 9.2) | 0.540 | -3.7 (-21.5, 14.1) | 0.683 | 0.491 |  |
| Children with suspected pneumonia receiving antibiotics | 0.2 (-16.0, 16.3) | 0.984 | -3.0 (-31.2, 25.2) | 0.835 | 23.3 (-9.6, 56.3) | 0.165 | 0.222 |  |
| Febrile children who received appropriate anti-malarial drugs | -1.2 (-16.7, 14.2) | 0.878 | 0.6 (-20.4, 21.6) | 0.958 | -20.9 (-36.7, -5.0) | 0.010 | 0.039 |  |
| Under-fives treatment score | -1.4 (-11.7, 8.9) | 0.791 | 3.0 (-8.1, 14.0) | 0.596 | -8.3 (-22.0, 5.3) | 0.232 | 0.453 |  |
| **Family planning** |  |  |  |  |  |  |  |  |
| Need for family planning satisfied | 6.6 (1.0, 12.2) | 0.020 | 7.4 (2.9, 11.8) | 0.001 | 4.2 (-1.5, 9.9) | 0.150 | 0.092 |  |
| Information on family planning provided | -1.3 (-6.3, 3.7) | 0.606 | -0.4 (-4.9, 4.2) | 0.880 | -4.3 (-10.1, 1.6) | 0.154 | 0.191 |  |
| Family planning score | 2.6 (-1.4, 6.5) | 0.197 | 3.5 (-0.1, 7.2) | 0.058 | 0.0 (-4.5, 4.4) | 0.987 | 0.747 |  |
| **Maternal health** |  |  |  |  |  |  |  |  |
| Antenatal clinic attendance at least four times | -10.5 (-23.1, 2.1) | 0.103 | -8.8 (-25.6, 8.1) | 0.306 | 5.6 (-7.6, 18.8) | 0.405 | 0.624 |  |
| Births attended by skilled caregiver | -3.2 (-11.1, 4.7) | 0.426 | 4.5 (-3.0, 12.0) | 0.240 | -1.8 (-10.0, 6.5) | 0.675 | 0.556 |  |
| Women attending postnatal care | 4.7 (-5.5, 15.0) | 0.366 | 4.6 (-3.9, 13.1) | 0.287 | 5.0 (-4.4, 14.5) | 0.297 | 0.276 |  |
| Maternal health score | -2.4 (-9.1, 4.3) | 0.481 | -0.1 (-6.1, 6.0) | 0.986 | 3.1 (-2.8, 9.0) | 0.303 | 0.269 |  |
| **Adult health** |  |  |  |  |  |  |  |  |
| Adults tested for HIV in last year | -3.0 (-7.1, 1.1) | 0.153 | -4.3 (-7.9, -0.6) | 0.022 | -2.1 (-7.3, 3.2) | 0.444 | 0.228 |  |
| TB suspects correctly screened for TB | 5.2 (-7.0, 17.4) | 0.402 | 3.1 (-16.6, 22.7) | 0.760 | 12.7 (-14.9, 40.4) | 0.367 | 0.433 |  |
| Ever hypertensive adults with currently controlled hypertension | 3.6 (-0.3, 7.5) | 0.073 | 2.5 (-1.7, 6.7) | 0.236 | 5.9 (0.9, 10.9) | 0.021 | 0.047 |  |
| Adult health score | 1.9 (-2.6, 6.4) | 0.404 | 0.1 (-4.8, 5.1) | 0.955 | 5.6 (-1.1, 12.2) | 0.100 | 0.200 |  |
| **Overall coverage score** | 1.4 (-2.1, 4.8) | 0.430 | 2.7 (-0.1, 5.4) | 0.060 | 1.7 (-1.9, 5.4) | 0.351 | 0.164 |  |

^1^Linear regression model of score at follow-up on score at baseline, district and time since cluster joined the intervention phase, with generalised estimating equations to give robust standard errors accounting for two measurements of each cluster.
